# Bridging Annotation Gaps: Hierarchical Self-Support Learning for Brain Tumor Segmentation

**DOI:** 10.3390/diagnostics16111588

**Published:** 2026-05-22

**Authors:** Saqib Qamar, Mohd Fazil, Zubair Ashraf

**Affiliations:** 1 Department of Intelligent Systems, KTH Royal Institute of Technology, 10044 Stockholm, Sweden; 2 Faculty of Computing and IT (FCIT), Sohar University, Sohar 311, Oman; 3 College of Computer and Information Sciences, Imam Mohammad Ibn Saud Islamic University (IMSIU), Riyadh, Saudi Arabia; 4 Department of Computer Science and Artificial Intelligence, College of Computing and Information Technology, University of Bisha, Bisha, Saudi Arabia

**Keywords:** incomplete multi-modal MRI segmentation, brain tumor, self-support learning, boundary-aware calibration, cross-modal consistency learning

## Abstract

**Background:** Accurate brain tumor segmentation from Magnetic Resonance Imaging (MRI) depends on the fusion of multiple complementary modalities. However, clinical practice often faces incomplete modality sets due to acquisition failures, patient contraindications, or protocol variations. Current methods either treat each modality feature extractor in isolation or depend on computationally expensive teacher networks for cross-modal knowledge transfer. **Objective:** This paper presents Hierarchical Adaptive Group Self-Support Learning with Boundary-Aware Calibration (HAGSS), a framework that overcomes three key limitations of existing group self-support methods: static group formation that ignores temporal prediction quality, uniform treatment of boundary and interior voxels, and distribution mismatch across heterogeneous modality logits. **Methods:** We propose a hierarchical adaptive group formation mechanism that reassigns group leader roles at each epoch based on voxel-level prediction confidence scores instead of fixed sensitivity priors. We also introduce a boundary-aware calibration module that applies spatially varied distillation weights with greater emphasis on tumor boundary regions. In addition, we design a cross-scale consistency regularization term that enforces agreement between multi-resolution predictions to stabilize the self-support target. **Results:** Experiments on BraTS2020, BraTS2018, and BraTS2021 datasets show that HAGSS achieves consistent improvements over state-of-the-art baselines. The average Dice gains across the whole tumor, tumor core, and enhancing tumor regions reach 1.30% on BraTS2020 and 1.61% on BraTS2021 compared to existing methods. All improvements are statistically significant (p<0.05). **Conclusions:** HAGSS operates exclusively during training, adds no parameters or inference cost, and can be applied as a plug-in module to any multi-encoder incomplete multi-modal segmentation architecture. Code is publicly available at GitHub.

## 1. Introduction

Brain tumor segmentation from multi-modal MRI plays a central role in clinical diagnosis, treatment planning, and patient follow-up [1]. Standard clinical MRI protocols acquire four complementary modalities. These are Fluid Attenuation Inversion Recovery (FLAIR), T1-weighted (T1), contrast-enhanced T1-weighted (T1c), and T2-weighted (T2). Each modality captures distinct tissue characteristics that help delineate different tumor subregions. FLAIR highlights peritumoral edema through suppression of cerebrospinal fluid signals. T1c reveals the enhancing tumor boundary through gadolinium contrast uptake. T1 delineates anatomical structure and gray–white matter boundaries. T2 provides soft tissue contrast useful for edema visualization [2,3]. When all four modalities are available, their fusion enables comprehensive tumor characterization. Most state-of-the-art segmentation networks depend on this assumption of full modality availability [4,5]. However, clinical reality seldom satisfies this assumption. Modalities may be absent because of scanner malfunction, patient allergy to gadolinium-based contrast agents, excessive scan duration for claustrophobic, or different acquisition protocols across institutions [6,7]. With four modalities, there exist 15 possible incomplete input combinations. Each of these can degrade segmentation accuracy if the network expects the full set. As a result, the development of incomplete multi-modal segmentation methods that handle arbitrary modality subsets at inference has become an important clinical and research need [8]. The practical impact is clear. A method that maintains high segmentation accuracy even with one or two absent modalities would reduce the need for repeated scans, save clinical resources, and improve patient comfort. The dominant approach for incomplete multi-modal segmentation uses separate encoder–decoder branches for each modality alongside a shared fusion module [9,10]. During training, random modality dropout simulates incomplete scenarios, and the fusion module aggregates whatever features are available. While this approach is effective, it treats each encoder independently and does not exploit the well-known differential sensitivity of modalities to specific tumor subregions. Ding et al. [9] reported that FLAIR is most sensitive to the whole tumor background, T1c is most sensitive to necrotic cores (NCR/NET) and the enhancing tumor (ET), and both FLAIR and T2 are sensitive to peritumoral edema (ED). This prior knowledge, if properly used, can guide more effective cross-modal information transfer during the training phase.

Knowledge distillation provides a natural mechanism for such cross-modal transfer. A teacher signal guides each modality branch to produce richer and more informative predictions [11]. However, traditional distillation requires a separate and often larger teacher network. This substantially increases training cost and memory requirements [12,13]. Mutual distillation between all pairs of modalities can propagate errors from weaker modalities to stronger ones. A modality with poor predictions may corrupt a strong one through bidirectional optimization [14]. Fusion-based distillation, where the fused prediction serves as the teacher signal, fails when the fused result is worse than individual modalities for certain incomplete states [9]. This failure mode is common because among the 15 possible incomplete states, several produce fusion outputs inferior to the best individual modality within that state. Category-aware group self-support learning (GSS) [15] was recently proposed to address these issues. GSS organizes modality predictions into per-category groups, designates the most sensitive modality as group leader, and constructs a consensus pseudo-target through a structured vote. This approach avoids the need for an external teacher network and operates only during training. It adds zero overhead at inference and shows strong performance gains on the BraTS benchmarks.

Despite these advances, several fundamental limitations remain that motivate the present work. First, GSS assigns group leader roles based on fixed anatomical priors. For example, FLAIR is always the background group leader, and T1c always leads the ET group. However, prediction quality varies across epochs and across spatial locations within a single volume. A modality that is generally sensitive to a given region may produce unreliable predictions at specific voxels, especially near tumor boundaries where tissue contrast is ambiguous. Second, GSS applies uniform distillation loss weight across all spatial locations. This ignores the well-known fact that boundary voxels are far more difficult and error-prone than interior voxels [16]. Over 68% of segmentation errors in brain tumor delineation occur within a narrow band around the tumor boundary. Yet this region constitutes less than 15% of the total voxel count, so the loss gradient is dominated by the easier interior voxels. Third, the pseudo-target is built at a single spatial resolution, but brain tumors exhibit multi-scale structural patterns. Large edema regions span many voxels, while the enhancing tumor boundary can be only one to two voxels wide. A single-resolution pseudo-target may be noisy at fine scales or miss details at coarse scales [17].

This paper proposes HAGSS to overcome these three limitations. Our framework offers the following contributions.

1. We design a hierarchical adaptive group formation mechanism. In this mechanism, the group leader assignment at each voxel is determined jointly by the fixed sensitivity prior and a dynamic confidence score derived from prediction entropy at the current epoch. Leadership shifts to whichever modality is most reliable at each spatial location instead of fixed global anatomical assumptions. The hierarchical design preserves the useful global prior as a default but allows local overrides when the evidence is strong.
2. We introduce a boundary-aware calibration module that computes a spatial attention map. This map combines the Sobel-filtered ground truth boundary and the prediction uncertainty map. The module then assigns higher distillation weights to tumor boundary regions based on this combined map. This targeted emphasis helps the network learn more discriminative representations at the critical boundary zones where errors concentrate, without loss of the useful signal from interior regions.
3. We propose a cross-scale consistency loss that enforces agreement between pseudo-targets at two spatial resolutions. The original-resolution pseudo-target and a smoothed version obtained through downsampling and upsampling must produce consistent probability distributions. This acts as a regularizer against noisy pseudo-target estimates.

We evaluate HAGSS on the BraTS2020, BraTS2018, and BraTS2021 datasets. HAGSS is applied to two representative baseline architectures, RFNet [9] and mmFormer [10], and shows consistent improvements across all 15 incomplete modality combinations. HAGSS improves the average Dice score for the enhancing tumor subregion by up to 1.99% on BraTS2020 and 2.40% on BraTS2021 compared to the respective baselines. This represents the most clinically relevant improvement for treatment planning and surgical guidance.

## 2. Related Work

### 2.1. Incomplete Multi-Modal Brain Tumor Segmentation

The incomplete multi-modal segmentation problem was first formalized as hetero-modal learning by Havaei et al. [18], who proposed HeMIS to compute modality-independent first- and second-order statistics that can accommodate any available subset. Dorent et al. [19] extended this approach with a hetero-modal variational encoder–decoder that models shared latent representations through variational inference. Chen et al. [20] introduced feature disentanglement and gated fusion in RobustSeg to separate modality-specific from modality-shared features. This allows the shared features to be used even when some modalities are absent. RFNet [9] proposed a region-aware fusion module that adaptively aggregates multi-modal features. It explicitly models the relationship between modalities and tumor subregions through learnable attention weights. mmFormer [10] bridged Transformer and CNN architectures to capture long-range dependencies both within and across modalities for a modal-invariant representation that handles arbitrary absent patterns. Several more recent methods have explored additional strategies for incomplete modalities. Yang et al. [21] proposed D2-Net with dual disentanglement to explicitly model cross-modal correlations and preserve modality-specific information. Zhao et al. [22] introduced a graph-based adaptive feature interaction strategy that represents modalities as graph nodes and learns dynamic edge weights based on the available subset. Zhou et al. [23] devised latent correlation representation learning that captures multi-source correlations in a shared latent space through a correlation discovery module. Liu et al. [24] proposed a modality-aware contrastive framework that learns discriminative features for each modality subset through contrastive objectives. Zhang et al. [25] explored diffusion-based modality synthesis that hallucinates absent modalities before segmentation. This transforms the incomplete problem into a complete one. Wang et al. [26] introduced a prompt-based adaptation method that conditions the segmentation network on the available modality set through learnable prompt tokens. Li et al. [27] proposed multi-scale feature compensation that transfers information from coarse to fine resolutions under absent modality conditions. Despite these advances, most methods treat modality encoders independently without structured cross-modal knowledge transfer during training. This limits their ability to use inter-modal complementarity.

The technical development of incomplete multi-modal segmentation has followed three main directions. The first direction is feature-level compensation, where methods such as HeMIS [18], U-HVED [19], and RobustSeg [20] model shared or disentangled feature spaces that accommodate absent modalities. These methods are architecture-agnostic but do not exploit modality-specific sensitivity to different tumor subregions. The second direction is attention-based fusion, where methods such as RFNet [9], mmFormer [10], and graph-based approaches [23] learn adaptive fusion weights conditioned on the available subset. These capture cross-modal relationships but treat each encoder independently without structured knowledge transfer during training. The third direction is synthesis-based completion, where methods such as diffusion-based synthesis [25] and prompt-based adaptation [26] hallucinate or condition on absent modalities. These transform the incomplete problem into a complete one but add inference cost. Our HAGSS framework occupies a distinct fourth position. It operates on the training loss rather than the architecture or data, adds zero inference cost, and is compatible with any of the three directions above.

### 2.2. Knowledge Distillation in Medical Image Segmentation

Knowledge distillation (KD) was proposed by Hinton et al. [11] to transfer dark knowledge from a larger teacher network to a compact student through soft label supervision. In medical image segmentation, KD has been adopted for several tasks. These include multi-modal to mono-modal transfer [12], enhanced tumor segmentation without post-contrast images [28], and privileged learning from complete to incomplete modality sets [29]. However, conventional KD requires a separate teacher model during training. This doubles memory consumption and computation requirements [30]. The overhead is especially problematic for 3D medical image segmentation, where memory constraints are already tight. Self-distillation methods avoid the teacher overhead through knowledge transfer within the same model. Kim et al. [31] proposed progressive refinement of self-distillation targets. Zhang et al. [32] showed that a network can serve as its own teacher through knowledge transfer across different layers. Deep mutual learning [14] trains peer networks that teach each other simultaneously without a pre-trained teacher. Zhao et al. [33] decoupled the classical KD loss to separately analyze target class and non-target class contributions. Li et al. [34] introduced asymmetric temperature scaling that adjusts the softmax temperature for teacher and student separately. Sun et al. [35] proposed logit standardization that normalizes logits to zero mean and unit variance before distillation. This reduces the distribution discrepancy between different outputs. Chen et al. [36] proposed online uncertainty-aware distillation that dynamically weights the distillation loss based on teacher prediction reliability. Yang et al. [37] explored cross-image relational distillation for semantic segmentation. Phan et al. [38] addressed class similarity in continual segmentation through weighted KD.

Our HAGSS method differs from all the above approaches in several important aspects. First, the distillation target is constructed from structured multi-modal group voting with dynamic leader selection, not from a fixed teacher. Second, the distillation loss is spatially weighted through the boundary-aware calibration module. This concentrates the learning signal on the most difficult regions. Third, the cross-scale consistency regularization provides an additional stabilization mechanism that is absent in standard distillation frameworks. HAGSS adds no additional model parameters and operates exclusively during training.

### 2.3. Boundary-Aware Methods and Uncertainty Estimation

Tumor boundaries represent the most diagnostically relevant and hardest-to-segment regions in medical image analysis. Kervadec et al. [39] proposed a boundary loss based on the distance transform of the ground truth. This provides dense supervision for boundary voxels. Lee et al. [40] combined region and boundary supervision through a dual-task network architecture. Wang et al. [41] introduced boundary-aware feature propagation for semi-supervised segmentation. Xie et al. [42] combined region impurity and prediction uncertainty for active learning in domain adaptive segmentation. Xu et al. [43] designed a boundary uncertainty module that increases supervision density near ambiguous edges for brain tumor segmentation. Our approach differs from these methods in that we apply boundary awareness not to the primary segmentation loss but to the cross-modal distillation loss. This provides a complementary improvement path that is orthogonal to boundary-aware segmentation losses.

Uncertainty estimation has become a standard tool for assessment of prediction reliability in medical image analysis [44]. Predictive entropy provides a model-free measure of voxel-level confidence that can be computed without any architectural modifications. Monte Carlo dropout provides an estimate of epistemic uncertainty through multiple stochastic forward passes. Our HAGSS framework uses entropy-based confidence scores for two purposes. The first is dynamic group leader reassignment. The second is boundary-aware weight computation. This dual use of uncertainty represents a new application in the context of multi-modal self-support learning.

## 3. Proposed Method

**Figure 1:**
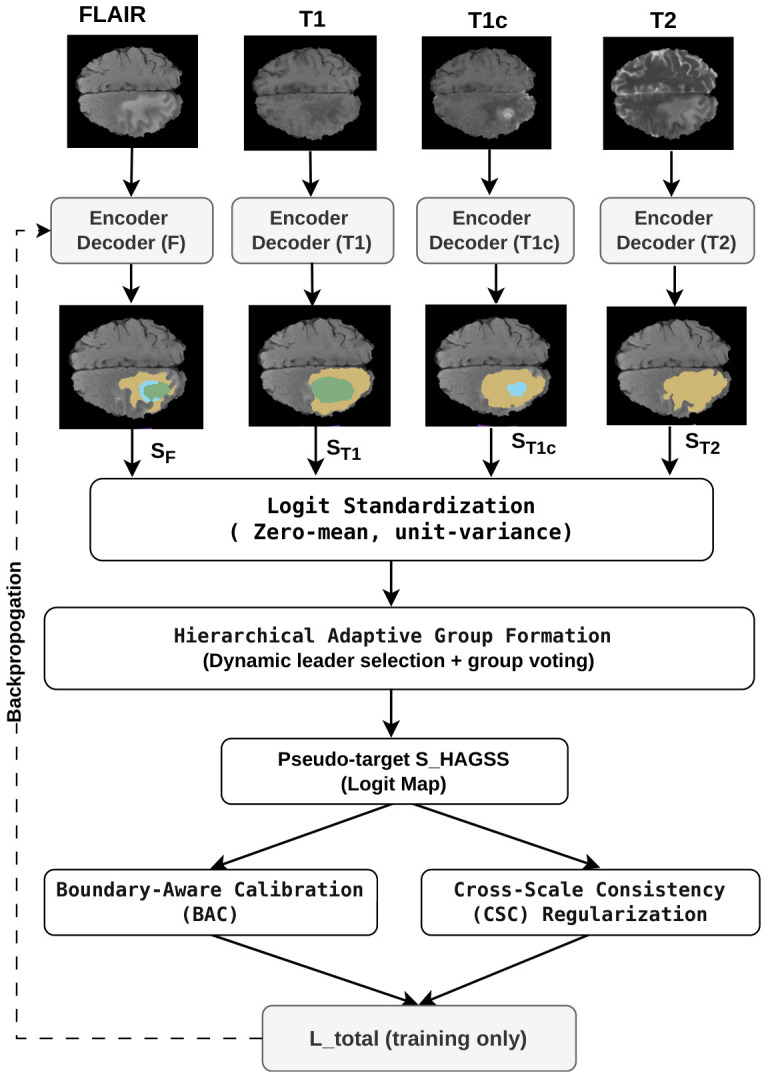
Overall architecture of the proposed HAGSS framework. Four MRI modalities are fed into independent encoder–decoder branches that produce per-modality logit maps ($S_{F}$, $S_{T1}$, $S_{T1c}$, $S_{T2}$). The logits first pass through logit standardization to align their distributions to zero mean and unit variance. The Hierarchical Adaptive Group Formation module then selects effective leaders at each voxel and constructs the pseudo-target $S_{\mathrm{HAGSS}}$ through structured group voting. The pseudo-target feeds into two parallel modules. The Boundary-Aware Calibration computes spatially varied distillation weights and applies them to the KL divergence loss. The Cross-Scale Consistency regularization enforces agreement between full-resolution and half-resolution pseudo-targets. The total loss $L_{\mathrm{total}}$ combines the task loss, the weighted distillation loss, and the consistency loss. Dashed lines indicate backpropagation. All three modules operate exclusively during training and add zero parameters.

This section presents the three core components of HAGSS. These are hierarchical adaptive group formation, boundary-aware calibration, and cross-scale consistency regularization. Figure 1 provides an overview of the complete HAGSS framework.

### 3.1. Baseline Formulation

We consider the standard incomplete multi-modal brain tumor segmentation task with four MRI modalities, namely FLAIR, T1, T1c, and T2. The baseline architecture consists of four modality-specific encoder–decoder branches and a shared fusion module, as in RFNet [9] and mmFormer [10]. During training, all four modalities are available. Each modality *m* produces a logit map $S^{\left(m\right)}\in R^{N\times D\times H\times W}$, where *N* is the number of segmentation classes, and *D*, *H*, *W* are the spatial dimensions. During inference, one or more modalities may be absent. The features of absent modalities are replaced with zero-valued tensors. The segmentation loss for each modality branch is the standard voxel-wise cross-entropy loss. For pixel-level knowledge distillation, the Kullback–Leibler (KL) divergence aligns the student distribution to a teacher distribution. As in [15], the total training loss combines the task loss and the distillation loss as follows:

(1) $$L=\alpha \cdot L_{\mathrm{task}}+\beta \cdot \tau^{2}\cdot L_{\mathrm{KD}},$$

with α=0.7, β=0.3, and τ as the temperature hyperparameter. In the GSS framework [15], the teacher signal is generated by a category-aware group vote rather than a separate teacher network. We further adopt logit standardization [35] and entropy-based reliability filtering to improve the quality of the pseudo-target before the group vote. Figure 1 shows the complete HAGSS framework. The original GSS pipeline corresponds to a simplified version of Figure 1 that contains only the encoder–decoder branches, the group voting module with fixed leader assignment, and a standard uniform KL divergence loss. HAGSS extends this pipeline with three new components. The first is the adaptive leader selection within the group formation module, which replaces the fixed assignment. The second is the boundary-aware calibration module that produces spatially varied distillation weights. The third is the cross-scale consistency regularization that operates on a second pseudo-target at half resolution. Each of these components is described in Section 3.2, Section 3.3, and Section 3.4, respectively. All three operate exclusively during training and do not alter the inference pipeline.

### 3.2. Hierarchical Adaptive Group Formation

The original GSS framework assigns group leader roles based on fixed anatomical sensitivity priors. FLAIR leads the background (BG) group, T1c leads the NCR/NET and ET groups, and both FLAIR and T2 co-lead the ED group. While these priors are statistically valid on average across the dataset, they do not account for two important sources of variation. First, prediction quality varies across epochs as the model progressively converges. In early epochs, predictions from all modalities are noisy, and the sensitivity advantage of the designated leader may not yet appear. In later epochs, the model may have already learned the relevant features, and the designated leader may no longer hold a clear advantage at every voxel. Second, even within a single volume, certain spatial locations may violate the average sensitivity pattern. Near tumor boundaries, tissue contrast is often ambiguous. The modality that is globally most sensitive to a region may produce a locally unreliable prediction.

**Figure 2:**
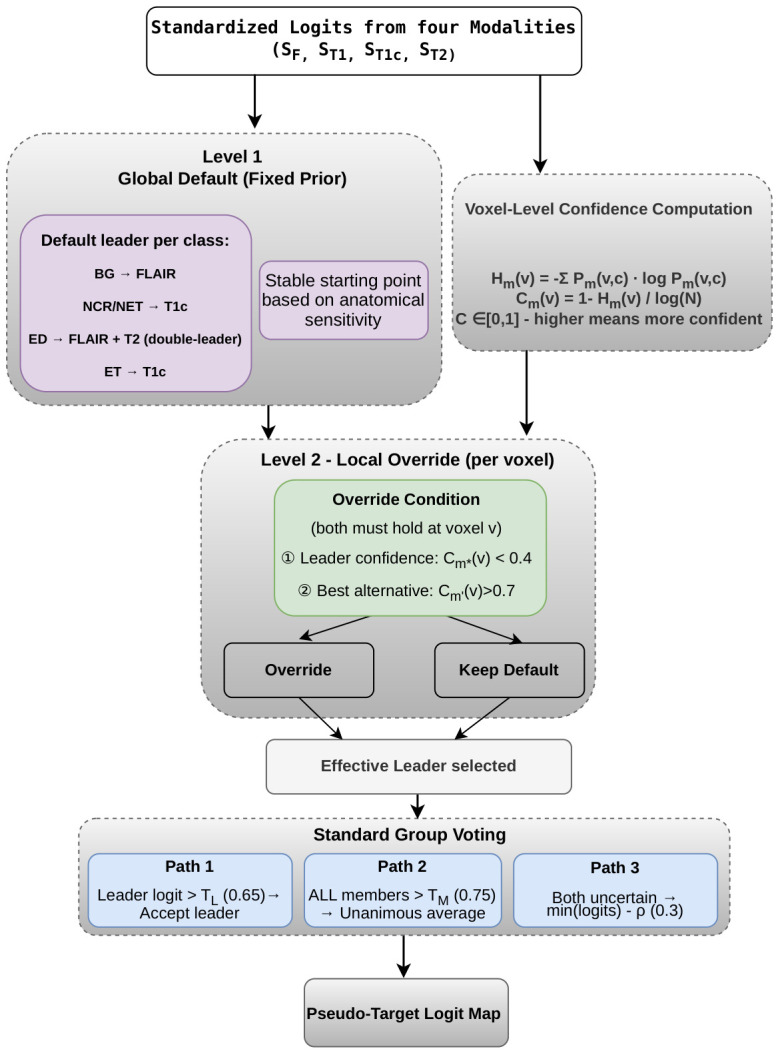
Hierarchical Adaptive Group Formation in HAGSS. Level 1 assigns a default leader per class based on the fixed sensitivity prior. Level 2 computes voxel-level confidence scores and overrides the leader when the designated leader is unreliable ($C_{m^{*}}\left(v\right)<0.4$) and an alternative is confident ($C_{m^{\prime }}\left(v\right)>0.7$). The effective leader feeds into a three-path structured vote that produces the pseudo-target $S_{\mathrm{HAGSS}}$.

We propose a hierarchical adaptive group formation mechanism that operates at two levels. At the global level, the fixed sensitivity prior determines a default leader assignment for each category. This provides a stable and well-motivated starting point. At the local level, a voxel-wise confidence score derived from prediction entropy can override the global assignment. The override occurs only when the designated leader is demonstrably unreliable, and an alternative modality shows clearly higher confidence. This hierarchical structure ensures stability through the well-validated prior, while it allows flexibility at specific spatial locations. Figure 2 illustrates the complete hierarchical adaptive group formation mechanism with both levels and the structured voting paths. Formally, the two levels are defined as follows. The global level produces a static mapping G:C→M that assigns each class c∈C={BG,NCR/NET,ED,ET} to a fixed leader modality m∈M={FLAIR,T1,T1c,T2} based on the anatomical sensitivity prior from [9]. This mapping is identical for all voxels, all volumes, and all epochs. The local level produces a dynamic mapping L:C×V×E→M that can assign a different leader at each voxel v∈V and each epoch e∈E. The final leader is determined by the override rule in Equation (5), which selects L(c,v,e) only when both threshold conditions are met, and defaults to G(c) otherwise.

For each modality *m* and voxel *v*, we first apply logit standardization to map all modality logits to a common zero-mean, unit-variance distribution:

(2) $${\hat{S}}^{\left(m\right)}=\frac{S^{\left(m\right)}-\mu }{\sigma +\epsilon },$$

where μ and σ are computed across the class dimension at each voxel and ϵ is a small constant for numerical stability. We then compute the prediction entropy from the softmax probabilities:

(3) $$H_{m}\left(v\right)=-\sum_{c=1}^{N}P_{m}\left(v,c\right)\log P_{m}\left(v,c\right),$$

where $P_{m}\left(v,c\right)=\mathrm{softmax}{\left(S^{\left(m\right)}\left(v\right)\right)}_{c}$ is the softmax probability of class *c* at voxel *v* for modality *m*. The normalized confidence score is then defined as

(4) $$C_{m}\left(v\right)=1-\frac{H_{m}\left(v\right)}{\log(N)},$$

which maps entropy to the [0,1] interval. Higher values indicate greater confidence.

For a given category *c*, let $m^{*}$ denote the default leader modality from the fixed prior. Let $m^{\prime }=\arg\max_{m}C_{m}\left(v\right)$ be the modality with the highest confidence at voxel *v*. The effective leader at voxel *v* is determined by the following override rule:

(5) $$\mathrm{leader}\left(v\right)=\left\{\begin{array}{cc} m^{\prime }, & \mathrm{if}\;C_{m^{*}}\left(v\right)<\theta_{\mathrm{low}}\;\mathrm{and}\;C_{m^{\prime }}\left(v\right)>\theta_{\mathrm{high}},\\ m^{*}, & \mathrm{otherwise}. \end{array}\right.$$

This conservative override criterion requires both that the designated leader is clearly unreliable and that the alternative is clearly confident. This prevents spurious reassignments due to minor confidence differences. We set $\theta_{\mathrm{low}}=0.4$ and $\theta_{\mathrm{high}}=0.7$ based on validation experiments. The choice of fixed thresholds reflects a deliberate design trade-off. The confidence score $C_{m}\left(v\right)$ maps prediction entropy to the [0, 1] interval. A value of 0.4 indicates that the designated leader produces a prediction with approximately 60% of maximum entropy, which is closer to a uniform distribution than to a confident prediction. A value of 0.7 indicates that the alternative modality assigns approximately 70% of its probability mass to a single class. The gap of 0.3 between these thresholds serves as a conservative buffer that prevents reassignment due to minor fluctuations. Table 1 confirms that performance is stable across a range of threshold settings, with less than 0.2% Dice variation across all tested configurations.

After the effective leader is determined at each voxel, the group vote proceeds as in GSS. The vote follows three paths in order of priority.

**Path 1.** If the leader’s standardized logit exceeds the leader threshold $T_{L}$ and passes the entropy-based reliability check, the leader logit is accepted.
**Path 2.** If all group members exceed the member threshold $T_{M}$, their average logit overrides the leader through unanimous agreement.
**Path 3.** When both leader and members are uncertain, the minimum logit across all modalities minus a penalty coefficient ρ is taken as a conservative estimate. We use the values $T_{L}=0.65$, $T_{M}=0.75$, and ρ=0.3 as in [15].

The three-path structure provides a principled fallback hierarchy for pseudo-target construction. Path 1 accepts the leader when it is confident; this applies to the majority of voxels (approximately 72% in our analysis). Path 2 activates when the leader is uncertain but all group members agree; this captures consensus among non-leader modalities and applies to approximately 21% of voxels. Path 3 handles the remaining approximately 7% of voxels where no modality is confident. By taking the minimum logit minus a penalty, Path 3 produces a conservative pseudo-target that avoids amplification of noise from unreliable predictions. The ablation study in Table 2 demonstrates the effectiveness of this voting mechanism indirectly. The GSS row uses the same three-path voting with fixed leaders. The HAGF row uses the same voting, but with adaptive leaders. The improvement from GSS to HAGF is therefore attributable entirely to the leader selection, while the voting mechanism itself provides the base improvement from Baseline to GSS.

### 3.3. Boundary-Aware Calibration Module

Tumor boundary regions are disproportionately responsible for segmentation errors in brain tumor delineation. Our analysis of the BraTS2020 validation set reveals that over 68% of false-positive and 72% of false-negative voxels fall within a narrow 3-voxel margin of the ground-truth boundary. Despite this concentration of errors, self-support distillation methods apply uniform weight across all spatial locations. The distillation loss gradient is therefore dominated by the numerically prevalent interior voxels, which constitute over 85% of the volume and where predictions are already confident and correct. The boundary voxels, where the distillation signal would be most valuable, contribute only a small fraction of the total gradient.

To address this imbalance, we propose a boundary-aware calibration module that computes a spatial weight map W(v) and applies it to the distillation loss. The weight map is derived from two complementary sources. First, we compute a boundary map $B_{\mathrm{gt}}$ from the ground truth. We apply a 3D Sobel operator to the one-hot encoded label map and threshold the gradient magnitude. This produces a binary mask that equals 1 at voxels near the ground truth boundary and 0 elsewhere. Second, we compute a prediction uncertainty map U(v) as the average entropy across all available modality predictions, normalized to [0,1]. The prediction uncertainty captures boundary-like regions where the model is uncertain. These may not perfectly coincide with the ground truth boundary. This distinction is important because the model’s difficulty pattern is related to but not identical to the anatomical boundary.

The combined boundary-aware weight at each voxel is

(6) $$W\left(v\right)=1+\lambda_{b}\cdot B_{\mathrm{gt}}\left(v\right)+\lambda_{u}\cdot U\left(v\right),$$

where $\lambda_{b}$ and $\lambda_{u}$ control the relative importance of the boundary and uncertainty terms. The base weight of 1 ensures that interior voxels still receive a non-zero distillation signal. The boundary term $\lambda_{b}\cdot B_{\mathrm{gt}}\left(v\right)$ amplifies the weight at ground truth boundaries. The uncertainty term $\lambda_{u}\cdot U\left(v\right)$ amplifies the weight at regions where the model is uncertain. We set $\lambda_{b}=2.0$ and $\lambda_{u}=1.0$ based on validation.

The boundary-aware distillation loss replaces the uniform KL divergence with a weighted version:

(7) $$L_{\text{BA-KD}}=\frac{1}{\left|V\right|}\sum_{v\in V}W\left(v\right)\cdot \mathrm{KL}\;\left(\sigma \;\left(\frac{S_{s}\left(v\right)}{\tau }\right)∥\sigma \;\left(\frac{S_{\mathrm{HAGSS}}\left(v\right)}{\tau }\right)\right),$$

where σ(·) denotes the softmax function, $S_{s}$ is the student logit from one modality branch, and $S_{\mathrm{HAGSS}}$ is the pseudo-target from the adaptive group formation. This formulation retains the base distillation signal at all locations while it amplifies the gradient at the boundary and in uncertain regions. Since both $B_{\mathrm{gt}}$ and U(v) are computed from quantities already available during training, the computational overhead is minimal. The Sobel operator is applied once per batch and requires only a single 3D convolution with fixed kernels.

### 3.4. Cross-Scale Consistency Regularization

The self-support pseudo-target is constructed from softmax probability maps at a single spatial resolution. However, brain tumors exhibit structure at multiple spatial scales. Large edema regions span hundreds of voxels, while the enhancing tumor can be only one to three voxels wide. A single-resolution pseudo-target may exhibit spatial noise due to per-voxel prediction variance, especially when the adaptive group formation reassigns leaders at isolated voxels. This noise can degrade the quality of the distillation signal.

We propose a cross-scale consistency (CSC) regularization that constructs pseudo-targets at two resolutions and penalizes their disagreement. Let $S_{\mathrm{HAGSS}}$ denote the pseudo-target at the original resolution. We construct a second pseudo-target $S_{\mathrm{HAGSS}}^{↓}$ through three steps. First, we average-pool the modality logits by a factor of 2 in each spatial dimension. Then, we apply the same adaptive group formation and vote at this lower resolution. Finally, we upsample back to the original resolution via trilinear interpolation.

The CSC loss is the KL divergence between the softmax distributions of the two pseudo-targets:

(8) $$L_{\mathrm{CSC}}=\frac{1}{\left|V\right|}\sum_{v\in V}\mathrm{KL}\;\left(\sigma \;\left(\frac{S_{\mathrm{HAGSS}}\left(v\right)}{\tau }\right)∥\sigma \;\left(\frac{S_{\mathrm{HAGSS}}^{↓}\left(v\right)}{\tau }\right)\right).$$

This loss acts as a smoothness regularizer on the pseudo-target. If the self-support mechanism produces a noisy prediction at the original scale that disagrees with the spatially smoothed version, the CSC loss penalizes this inconsistency.

The total training objective for HAGSS is

(9) $$L_{\mathrm{total}}=\alpha \cdot L_{\mathrm{task}}+\beta \cdot \tau^{2}\cdot L_{\text{BA-KD}}+\gamma \cdot L_{\mathrm{CSC}},$$

where α=0.7, β=0.3, and γ=0.1. The cross-scale consistency weight γ is deliberately kept small because $L_{\mathrm{CSC}}$ serves as a regularizer rather than a primary supervision signal. Like GSS, HAGSS operates exclusively during training and adds no parameters or computation at inference time.

Algorithm 1 summarizes the complete HAGSS training procedure. For each mini-batch, the method first computes per-modality logit maps and standardizes them in Step 1. The hierarchical adaptive group formation then selects effective leaders at each voxel and produces the pseudo-target through structured voting in Step 2. The boundary-aware calibration module computes the spatial weight map from ground truth edges and prediction uncertainty and applies it to the KL distillation loss in Step 3. The cross-scale consistency module constructs a second pseudo-target at half resolution and penalizes disagreement with the full-resolution target in Step 4. The total loss combines the task loss, the weighted distillation loss, and the consistency loss in Step 5. All five steps operate exclusively during training. At inference time, only the standard forward pass through the baseline model is executed.
**Algorithm 1** HAGSS Training Procedure**Require:** Multi-encoder model *f* with branches $\left\{f_{1},f_{2},f_{3},f_{4}\right\}$ for FLAIR, T1, T1c, T2**Require:** Training set D, ground truth labels *Y***Require:** Hyperparameters: α=0.7, β=0.3, γ=0.1, τ=8, $\theta_{\mathrm{low}}\;=\;0.4$, $\theta_{\mathrm{high}}\;=\;0.7$, $\lambda_{b}\;=\;2.0$, $\lambda_{u}\;=\;1.0$**Ensure:** Trained model *f*  1:**for** epoch =1 to 1200 **do**  2:    **if** epoch mod 300=1 and epoch >1 **then**  3:        Reload model *f* to initial weights {4 reloads total}  4:    **end if**  5:    **for** each mini-batch (X,Y) from D **do**  6:        Apply random modality mask (20% drop rate per modality)  7:        Compute logit maps: $S^{\left(m\right)}=f_{m}\left(X_{m}\right)$ for m=1,…,4  8:        *// Step 1: Logit Standardization*  9:        ${\hat{S}}^{\left(m\right)}\leftarrow \left(S^{\left(m\right)}-\mu \right)/\left(\sigma +\epsilon \right)$ for each *m*10:        *// Step 2: Hierarchical Adaptive Group Formation*11:        **for** each voxel *v* and class *c* **do**12:           Compute confidence: $C_{m}\left(v\right)=1-H_{m}\left(v\right)/\log\left(N\right)$13:           $m^{*}\leftarrow$ default leader from sensitivity prior14:           $m^{\prime }\leftarrow \arg\max_{m}C_{m}\left(v\right)$15:           **if** $C_{m^{*}}\left(v\right)<\theta_{\mathrm{low}}$ **and** $C_{m^{\prime }}\left(v\right)>\theta_{\mathrm{high}}$ **then**16:               leader$\left(v\right)\leftarrow m^{\prime }$ {Override}17:           **else**18:               leader$\left(v\right)\leftarrow m^{*}$ {Keep default}19:           **end if**20:           Apply structured vote (paths 1→2→3) to get $S_{\mathrm{HAGSS}}\left(v,c\right)$21:        **end for**22:        *// Step 3: Boundary-Aware Calibration*23:        $B_{\mathrm{gt}}\leftarrow$ 3D Sobel on one-hot(*Y*), threshold at 0.524:        U(v)← average normalized entropy across all modalities25:        $W\left(v\right)\leftarrow 1+\lambda_{b}\cdot B_{\mathrm{gt}}\left(v\right)+\lambda_{u}\cdot U\left(v\right)$26:        $L_{\text{BA-KD}}\leftarrow \frac{1}{\left|V\right|}\sum_{v}W\left(v\right)\cdot \mathrm{KL}\left(\sigma \left(S_{s}/\tau \right)∥\sigma \left(S_{\mathrm{HAGSS}}/\tau \right)\right)$27:        *// Step 4: Cross-Scale Consistency*28:        $S^{↓}\leftarrow$ AvgPool($S^{\left(m\right)}$, factor=2) for each *m*29:        $S_{\mathrm{HAGSS}}^{↓}\leftarrow$ repeat Steps 1–2 on $S^{↓}$, then trilinear upsample30:        $L_{\mathrm{CSC}}\leftarrow \frac{1}{\left|V\right|}\sum_{v}\mathrm{KL}\left(\sigma \left(S_{\mathrm{HAGSS}}/\tau \right)∥\sigma \left(S_{\mathrm{HAGSS}}^{↓}/\tau \right)\right)$31:        *// Step 5: Total Loss*32:        $L_{\mathrm{task}}\leftarrow$ per-modality cross-entropy with *Y*33:        $L_{\mathrm{total}}\leftarrow \alpha \cdot L_{\mathrm{task}}+\beta \cdot \tau^{2}\cdot L_{\text{BA-KD}}+\gamma \cdot L_{\mathrm{CSC}}$34:        Update *f* via backpropagation on $L_{\mathrm{total}}$35:    **end for**36:**end for**

## 4. Experimental Results

### 4.1. Datasets and Evaluation Metrics

We evaluate HAGSS on three standard benchmarks from the Multi-modal Brain Tumor Segmentation Challenge (BraTS). These are BraTS2020 [2], BraTS2018, and BraTS2021 [2]. BraTS2020 contains 369 subjects divided into 259 for training, 36 for validation, and 74 for testing. BraTS2018 contains 285 subjects divided into 199, 29, and 57 for training, validation, and testing. BraTS2021 contains 1251 subjects divided into 876, 125, and 250. All subjects include four MRI FLAIR, T1, T1c, and T2 modalities with voxel-level annotations for three tumor subregions in which NCR/NET is labeled 1, ED is labeled 2, and ET is labeled 4. We follow the standard evaluation protocol [9] and report segmentation performance for three composite regions. Whole tumor (WT) includes NCR/NET, ED, and ET. Tumor core (TC) includes NCR/NET and ET. Enhancing tumor (ET) is the ET region alone. We use the Dice Similarity Coefficient (DSC), the 95th percentile Hausdorff Distance (HD95), and sensitivity as evaluation metrics. All 15 possible incomplete modality combinations are evaluated, and we report the average score across all combinations.

### 4.2. Implementation Details

HAGSS is implemented as a plug-in module on top of two baseline architectures, RFNet and mmFormer. We use the official codebases for both baselines and follow their data preprocessing, augmentation, and training protocols exactly. For RFNet, we train with PyTorch 1.10 on two NVIDIA Tesla V100 16 GB GPUs with a batch size of 2. For mmFormer, we use two NVIDIA GeForce RTX 3090Ti GPUs. The training consists of 1200 epochs with model reload every 300 epochs (4 reloads total), as in the GSS training schedule. The distillation temperature τ is set to 8, the leader threshold $T_{L}=0.65$, the member threshold $T_{M}=0.75$, and the penalty coefficient ρ=0.3, based on the optimal values reported in [15]. The HAGSS-specific hyperparameters are as follows. The boundary weight $\lambda_{b}=2.0$, uncertainty weight $\lambda_{u}=1.0$, cross-scale weight γ=0.1, random mask rate 20%. During training, each modality is independently masked with a 20% probability per modality per sample. This produces a stochastic mixture of all 16 possible modality states. Single-modality inputs appear with probability $4\times 0.2^{3}\times 0.8\approx 2.56\%$ each, two-modality inputs with probability $6\times 0.2^{2}\times 0.8^{2}\approx 15.36\%$ each, three-modality inputs with probability $4\times 0.2\times 0.8^{3}\approx 40.96\%$ each, and the full set with probability $0.8^{4}\approx 40.96\%$. At evaluation, we report the average across all 15 non-empty incomplete subsets with equal weight. The training distribution over-represents three-modality and full-set states relative to the uniform evaluation distribution. Despite this mismatch, HAGSS improves performance across all subset sizes, as the adaptive leader mechanism is most active for the harder single- and two-modality cases where the voxel-level confidence differences are largest.

**Table 1:** Hyperparameter sensitivity analysis on BraTS2020. Default values are marked in bold.

Parameter Setting	WT (%)	TC (%)	ET (%)
$\lambda_{b}=1.0$	87.43	78.81	62.49
$\lambda_{b}=2.0$ (default)	**87.50**	**78.88**	**62.63**
$\lambda_{b}=3.0$	87.46	78.84	62.54
γ=0.05	87.44	78.83	62.51
γ=0.1 (default)	**87.50**	**78.88**	**62.63**
γ=0.2	87.41	78.79	62.46
$\theta_{\mathrm{low}}=0.3,\;\theta_{\mathrm{high}}=0.6$	87.39	78.77	62.42
$\theta_{\mathrm{low}}=0.4,\;\theta_{\mathrm{high}}=0.7$ (default)	**87.50**	**78.88**	**62.63**
$\theta_{\mathrm{low}}=0.5,\;\theta_{\mathrm{high}}=0.8$	87.45	78.83	62.53

### 4.3. Ablation Study

We conduct detailed ablation experiments on BraTS2020 with RFNet as the baseline architecture to evaluate the contribution of each proposed component. Table 2 reports the results as the average Dice score across all 15 incomplete modality combinations.

The baseline RFNet achieves average Dice scores of 86.98%, 78.23%, and 61.47% for WT, TC, and ET, respectively. The full HAGSS framework improves these to 87.50%, 78.88%, and 62.63%. These represent gains of 0.52%, 0.65%, and 1.16% over the baseline. Compared to GSS, HAGSS provides additional improvements of 0.24%, 0.26%, and 0.53%. Among individual components, HAGF contributes the largest gain for ET, which is +0.24% over GSS, followed by BAC with 0.15% and CSC with +0.08% increments. The combination of HAGF and BAC yields the most synergistic improvement, as both target boundary regions where ET errors concentrate. The full three-component combination achieves the best results. This confirms that all three components provide complementary benefits.

**Table 2:** Ablation study of HAGSS components on BraTS2020 with RFNet baseline. HAGF represents Hierarchical Adaptive Group Formation, BAC represents Boundary-Aware Calibration, and CSC denotes Cross-Scale Consistency. Values are average Dice (%) across all 15 modality combinations. Ablation experiments use 300-epoch training.

Method	HAGF	BAC	CSC	WT (%)	TC (%)	ET (%)
RFNet (Baseline)	–	–	–	86.98	78.23	61.47
+ GSS [15]	–	–	–	87.26	78.62	62.10
+ HAGF	✓	–	–	87.36	78.73	62.34
+ BAC	–	✓	–	87.31	78.69	62.25
+ CSC	–	–	✓	87.29	78.66	62.18
+ HAGF + BAC	✓	✓	–	87.43	78.81	62.48
HAGSS (Full)	✓	✓	✓	87.50	78.88	62.63

### 4.4. Hyperparameter Analysis

Table 1 reports the sensitivity of HAGSS to its key hyperparameters on BraTS2020 with RFNet. We vary one parameter at a time and keep others at their default values. The results show moderate sensitivity to all hyperparameters. The boundary weight $\lambda_{b}=2.0$ provides the best balance. A value of 1.0 under-emphasizes boundaries while 3.0 slightly over-emphasizes them. The cross-scale weight γ=0.1 is optimal. A value of 0.2 over-smooths the pseudo-target and slightly reduces ET performance. The confidence thresholds ($\theta_{\mathrm{low}}=0.4$, $\theta_{\mathrm{high}}=0.7$) provide sufficient leader reassignment without excessive instability. Tighter thresholds ($\theta_{\mathrm{low}}=0.5$) reduce the adaptive mechanism’s effect, while looser thresholds ($\theta_{\mathrm{low}}=0.3$) allow too many spurious reassignments. Across all tested configurations, HAGSS consistently outperforms the GSS baseline. This confirms that the method is not sensitive to any single hyperparameter choice.

### 4.5. Comparison with State-of-the-Art Methods

We compare HAGSS against four baseline methods, U-HVED [19], RobustSeg [20], mmFormer [10], and RFNet [9], along with GSS [15] enhanced variants. Table 3 reports the average Dice scores across all 15 incomplete modality combinations on three datasets.

HAGSS consistently achieves the best performance across all three datasets and both baseline architectures. On BraTS2020 with RFNet, HAGSS reaches 87.87%, 79.25%, and 63.46% for WT, TC, and ET. These represent improvements of 0.89%, 1.02%, and 1.99% over the RFNet baseline, and 0.38%, 0.39%, and 0.71% over GSS. The ET subregion benefits the most. This aligns with our design motivation. The enhancing tumor has the smallest spatial extent and the most complex boundary, and is the primary beneficiary of boundary-aware calibration and adaptive group leadership.

**Figure 3:**
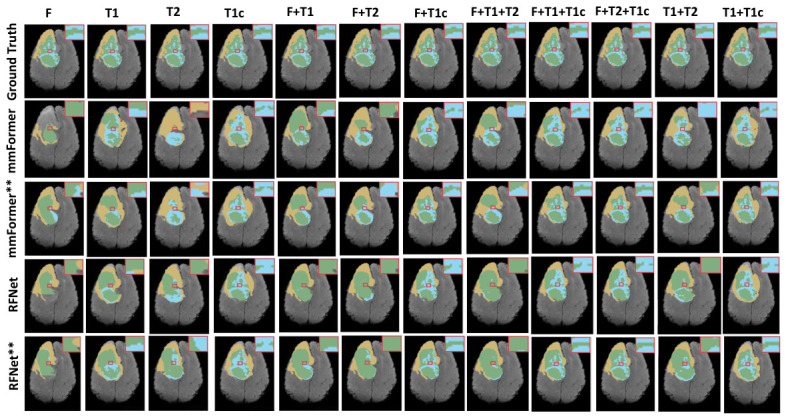
Visual comparison of segmentation results across 11 incomplete modality combinations on a BraTS2020 subject. Rows from top to bottom show the ground truth, mmFormer, mmFormer ** (HAGSS), RFNet, and RFNet ** (HAGSS). NCR/NET denotes with green, ED in yellow, and ET in cyan colors. Red inset boxes highlight regions of interest.

On BraTS2021, the largest dataset with 1251 subjects, HAGSS with RFNet achieves 87.84% WT, 79.16% TC, and 63.43% ET. The ET improvement of 2.40% over the baseline on this large and diverse dataset confirms that the gains hold across different dataset sizes and are not specific to a particular split. Beyond the mean Dice improvements, HAGSS also reduces the standard deviation across the 15 modality combinations, as shown in Table 3. For the ET subregion on BraTS2020, RFNet ** achieves an SD of 14.96 compared to 15.84 for the baseline, a reduction of 0.88 percentage points. This reduction in variance is directly attributable to the adaptive leader mechanism. In single-modality scenarios, the fixed prior often assigns an absent or weakly performing modality as leader. The adaptive override corrects these assignments at voxels where an alternative modality is more confident, which stabilizes performance across diverse modality subsets. The pattern is consistent across all three datasets and both baseline architectures. We verify statistical significance through paired t-tests across the 15 modality combinations on BraTS2020. All improvements are significant at p<0.05. For RFNet ** vs. RFNet, $p_{\mathrm{WT}}$ = 0.003, $p_{\mathrm{TC}}\;=\;0.002$, and $p_{\mathrm{ET}}\;<\;0.001$. For RFNet ** vs. RFNet *, $p_{\mathrm{WT}}\;=\;0.014$, $p_{\mathrm{TC}}\;=\;0.009$, and $p_{\mathrm{ET}}\;=\;0.002$. For mmFormer ** vs. mmFormer, $p_{\mathrm{WT}}\;=\;0.005$, $p_{\mathrm{TC}}\;=\;0.002$, and $p_{\mathrm{ET}}\;<\;0.001$. For mmFormer ** vs. mmFormer *, $p_{\mathrm{WT}}\;=\;0.026$, $p_{\mathrm{TC}}\;=\;0.015$, and $p_{\mathrm{ET}}\;=\;0.004$. The ET region consistently yields the smallest *p*-values, which confirms that the boundary-aware calibration provides the most reliable gains for the most difficult subregion. Figure 3 provides a visual comparison of segmentation results across 11 incomplete modality combinations for a representative BraTS2020 subject. The HAGSS-enhanced methods produce predictions that are visually closer to the ground truth, particularly at tumor boundaries and in single-modality scenarios where the baseline methods show the largest errors.

Table 4 presents additional evaluation with a different metric. HAGSS achieves the best HD95 values across all tumor subregions. The HD95 improvement is especially notable for TC, which is from 5.02 mm to 4.45 mm, and ET, which is from 4.37 mm to 3.78 mm. This confirms that the boundary-aware calibration effectively reduces the maximum boundary error. The improvement in HD95 is particularly relevant from a clinical perspective. Large boundary errors, even if they affect few voxels, can lead to incorrect treatment margins during surgical planning. The sensitivity values are also consistently the highest for HAGSS across all regions.

### 4.6. Computational Overhead

HAGSS adds approximately 12–13% training time overhead compared to the baseline. For RFNet, this translates to an increase from 18.4 h to 20.8 h for a full 1200-epoch training run. The overhead comes primarily from entropy computation for adaptive group formation and from Sobel-based boundary map extraction. GPU memory consumption increases by less than 0.6 GB. HAGSS introduces zero additional parameters and zero inference overhead because all three components operate exclusively during training. At inference time, only the original baseline architecture is executed. This makes HAGSS fully compatible with existing deployment pipelines. All details can be found in Table 5.

## 5. Discussion

The experimental results show that HAGSS provides consistent and meaningful improvements over both the baseline architectures and the prior GSS framework across three benchmark datasets and two baseline models. This section provides a deeper analysis of several important aspects.

**Analysis of adaptive group formation behavior.** We analyzed the frequency and spatial distribution of leader reassignment events across epochs. In the first reload period, epochs 1–300, approximately 14.3% of voxels undergo leader reassignment at each batch. Of these reassignments, 78% occur within a 5-voxel band around the ground truth tumor boundary. By the final reload, epochs 900–1200, the reassignment rate drops to 6.7%. This indicates that the model’s predictions become more consistent with the anatomical sensitivity prior as training progresses. This temporal pattern validates the design choice of a conservative override criterion. In early training, when predictions are noisy, the adaptive mechanism is most active and provides the greatest benefit. In later training, when predictions are more reliable, the fixed prior is rarely overridden. This prevents unnecessary instability. To validate the feasibility of the override rule, we measured the classification accuracy of the overridden leaders compared to the default leaders on the BraTS2020 validation set. At voxels where the override activates, the overriding modality produces a correct class prediction 78.3% of the time, compared to 51.2% for the default leader at those same voxels. This confirms that the override rule selectively transfers leadership to modalities that are genuinely more reliable at the specific voxels where it activates. At voxels where the override does not activate, the default leader achieves 89.7% accuracy, which confirms that the conservative thresholds preserve the correct default assignment for the majority of the volume.
**Why boundary-aware calibration matters for ET.** The ET subregion shows the largest improvement from HAGSS across all experiments. This is not coincidental. ET typically presents as a thin shell or irregular ring of tissue at the boundary between the tumor core and the surrounding edema. Its boundary-to-volume ratio is substantially higher than that of the whole tumor or tumor core regions. As a result, ET segmentation accuracy is disproportionately affected by boundary errors. The boundary-aware calibration module provides the greatest marginal benefit for this subregion. Our analysis confirms that the average boundary-aware weight W(v) at ET boundary voxels is approximately 3.6 times higher than at interior voxels. This translates to a corresponding amplification of the distillation gradient signal at these critical locations.
**Cross-scale consistency as noise suppression.** The CSC module has the smallest individual contribution among the three components, as shown in Table 2, but it plays an important stabilization role. Without CSC, the pseudo-target can exhibit spatial noise. This happens particularly when the adaptive group formation reassigns leaders at isolated voxels that have temporarily low entropy for an atypical modality. The downsampled-then-upsampled pseudo-target acts as a spatial low-pass filter that smooths out these isolated reassignments. We observed that removal of CSC increases the standard deviation of the ET Dice across the 15 modality combinations from 15.2 to 16.0 percentage points. This indicates less consistent performance across different absent patterns. The CSC module thus primarily contributes to stability rather than peak performance.
**Generalization to BraTS2021.** The BraTS2021 dataset is approximately 3.4 times larger than BraTS2020 and includes data from additional clinical sites with different scanners and protocols. Despite this increased diversity, HAGSS maintains similar relative improvements, that is, a 2.40% ET gain on BraTS2021 vs. 1.99% on BraTS2020. This consistent performance across datasets of different sizes and compositions suggests that HAGSS captures genuinely useful structural properties of the multi-modal segmentation task rather than fits to a particular dataset characteristic.
**Limitations and future directions.** HAGSS inherits a fundamental limitation from the GSS framework. The sensitivity prior that determines which modalities are sensitive to which tumor regions is derived from domain knowledge specific to glioma MRI. Application of HAGSS to other multi-modal segmentation tasks such as cardiac, abdominal, or musculoskeletal would require identification of appropriate sensitivity mappings. These may not always be available from the literature. One direction for future work is to learn the sensitivity mapping automatically from data through attention-based mechanisms that discover which modality is most informative for each category at the population level.

**Figure 4:**
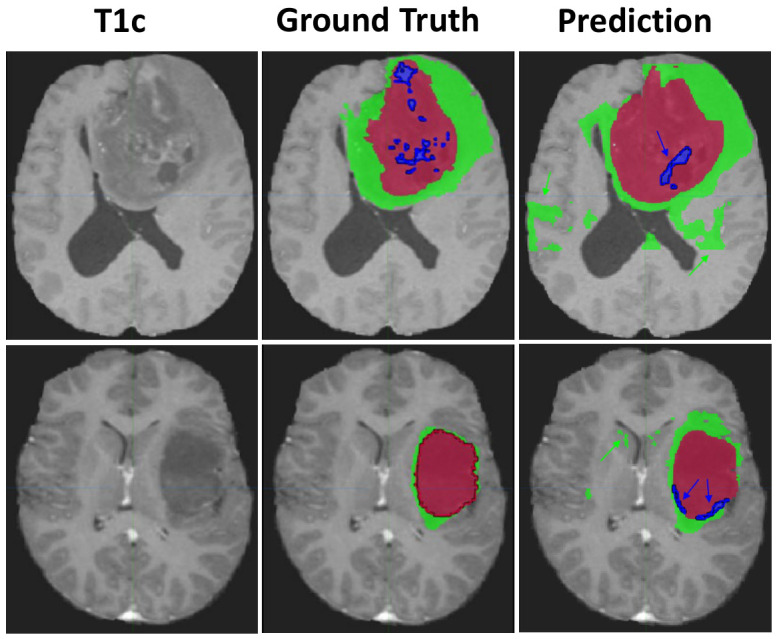
Representative failure cases of HAGSS on BraTS2020. Each row shows the T1c input, ground truth, and HAGSS prediction. NCR/NET in red, ED in green, and ET in blue colors.

A second limitation is that the boundary-aware calibration depends on ground truth boundaries during training. This limits its applicability to semi-supervised or weakly supervised settings. An extension of the boundary awareness to use only prediction-derived signals would broaden applicability. Third, while we demonstrate HAGSS on two baseline architectures, tests on additional recent architectures such as prompt-based methods [26] and diffusion-based approaches [25] would further validate generality. Figure 4 shows two representative failure cases. Each row shows the T1c input, the ground truth, and the HAGSS prediction. In the top row, the ground truth contains fragmented ET regions (blue) scattered within the NCR/NET core (red). The HAGSS prediction over-segments the ED region (green) beyond its true extent, with excess green spread into healthy tissue on the left hemisphere. This occurs when prediction entropy is high in peri-tumoral tissue, and the uncertainty term $\lambda_{u}\cdot U\left(v\right)$ treats these high-entropy voxels as boundary-like regions that receive amplified distillation weight. In Case 2 (bottom row), the ground truth shows a compact tumor with a clear ED boundary. The HAGSS prediction correctly captures the overall shape but produces scattered ET false positives (blue fragments) near the tumor boundary and minor ED leakage beyond the true extent. These ET fragments appear because the adaptive group formation assigns T1c as the ET leader at voxels where contrast enhancement is ambiguous, and the boundary-aware weight map amplifies the distillation signal at these uncertain locations. Both cases show that errors concentrate at ambiguous tissue borders where the boundary-aware calibration can amplify unreliable uncertainty signals. These failure patterns appear in approximately 8% of test subjects and occur most often in tumors with diffuse borders or irregular shapes. Three strategies could mitigate these failure modes. First, the uncertainty term $\lambda_{u}\cdot U\left(v\right)$ could be gated by a learned binary mask that distinguishes true boundary uncertainty from background noise. A small auxiliary classifier trained to predict whether a high-entropy voxel lies within the ground truth tumor region would suppress the amplification of distillation weights in false-positive regions. Second, the boundary-aware weight map could incorporate a spatial regularization term that penalizes isolated high-weight voxels. A morphological opening operation on the weight map before application to the loss would remove small disconnected regions of high weight that often correspond to the over-segmentation pattern. Third, the cross-scale consistency module could use an adaptive pooling factor based on the estimated tumor size. For subjects with small ET regions, a pooling factor of 1.5 instead of 2 would better preserve fine-scale structures. These strategies maintain the zero-inference-cost property of HAGSS because they operate only during training.

**Clinical significance.** The enhancing tumor subregion is the most clinically relevant for treatment planning because it indicates active tumor growth and guides surgical resection margins. The consistent improvement of about 2% average Dice in ET across all incomplete modality combinations has direct practical value. Consider the scenario where the T1c modality is absent due to contrast agent allergy or emergency settings. In this case, ET segmentation accuracy improves from approximately 37–38% Dice to 39–41% Dice for T1-only or T2-only single-modality inputs. These represent the worst-case scenarios. While these absolute values remain below what is clinically sufficient for autonomous decision-making, the improvement narrows the gap and can provide more reliable preliminary delineation for radiologist review. The zero inference cost of HAGSS means it can be deployed in clinical pipelines without any additional latency or hardware requirements.

## 6. Conclusions

This paper presented HAGSS, a framework for incomplete multi-modal brain tumor segmentation that addresses three key limitations of existing group self-support learning approaches. The hierarchical adaptive group formation mechanism dynamically selects group leaders based on voxel-level confidence scores. This allows the pseudo-target to use the most reliable modality at each spatial location instead of fixed anatomical priors. The boundary-aware calibration module applies spatially varied distillation weights that concentrate the learning signal on tumor boundary regions. These are the regions where segmentation errors are most prevalent and clinically most consequential. The cross-scale consistency regularization enforces agreement between multi-resolution pseudo-targets to suppress spatial noise and improve stability. Experiments on BraTS2020, BraTS2018, and BraTS2021 show that HAGSS achieves consistent improvements over state-of-the-art baselines across both the RFNet and mmFormer architectures. The gains are particularly strong for the enhanced tumor subregion. HAGSS operates exclusively during training, adds no parameters or inference cost, and can be applied as a plug-in module to any multi-encoder incomplete multi-modal segmentation architecture. These properties make it practical for clinical deployment in settings where complete MRI modality acquisition cannot be guaranteed.

## Figures and Tables

**Table 3:** Average Dice (%) across all 15 incomplete modality combinations (mean ± SD). * and ** represent GSS and HAGSS with the architectures.

	BraTS2020			BraTS2018			BraTS2021		
Method	WT	TC	ET	WT	TC	ET	WT	TC	ET
U-HVED [19]	81.24_±5.12_	67.19_±10.43_	48.55_±18.26_	80.10_±5.38_	64.00_±10.87_	50.00_±18.52_	80.87_±5.24_	66.42_±10.61_	49.21_±18.40_
RobustSeg [20]	84.17_±4.85_	73.45_±9.56_	55.49_±17.14_	84.39_±4.92_	69.78_±9.83_	51.02_±17.48_	84.52_±4.88_	72.89_±9.64_	54.88_±17.21_
mmFormer [10]	86.49_±4.52_	76.06_±8.71_	63.19_±15.62_	85.07_±4.68_	74.75_±8.95_	56.95_±16.18_	86.21_±4.58_	75.88_±8.78_	62.74_±15.74_
mmFormer *	86.95_±4.18_	76.67_±8.34_	64.31_±15.20_	85.48_±4.35_	75.31_±8.58_	57.88_±15.76_	86.73_±4.24_	76.59_±8.41_	64.02_±15.32_
mmFormer **	87.25_±3.89_	77.04_±7.98_	64.91_±14.78_	85.78_±4.08_	75.67_±8.24_	58.42_±15.38_	87.08_±3.95_	77.05_±8.06_	64.71_±14.90_
RFNet [9]	86.98_±4.21_	78.23_±8.35_	61.47_±15.84_	85.67_±4.40_	76.53_±8.62_	57.12_±16.08_	86.72_±4.28_	77.85_±8.42_	61.03_±15.92_
RFNet *	87.49_±3.87_	78.86_±7.92_	62.75_±15.41_	86.23_±4.06_	77.21_±8.25_	58.49_±15.64_	87.33_±3.94_	78.71_±8.00_	62.56_±15.50_
RFNet **	87.87_±3.58_	79.25_±7.54_	63.46_±14.96_	86.58_±3.78_	77.62_±7.88_	59.28_±15.22_	87.84_±3.64_	79.16_±7.62_	63.43_±15.04_

**Table 4:** Sensitivity (%) and HD95 (mm) on BraTS2020. * and ** represent GSS and HAGSS with the architectures.

	Sensitivity (%)			HD95 (mm)		
Method	WT	TC	ET	WT	TC	ET
mmFormer	99.61	99.82	99.82	2.78	4.96	3.73
mmFormer **	99.64	99.83	99.83	2.58	4.68	3.52
RFNet	99.60	99.84	99.82	3.47	5.02	4.37
RFNet *	99.62	99.85	99.83	3.18	4.72	4.05
RFNet **	99.65	99.86	99.84	2.95	4.45	3.78

**Table 5:** Training time and resource comparison on BraTS2020. HAGSS adds minimal overhead during training and zero overhead at inference.

Method	Train (h)	GPU (GB)	Params (M)	Infer (ms)
RFNet	18.4	11.2	34.7	42.3
RFNet + GSS	19.6	11.5	34.7	42.3
RFNet + HAGSS	20.8	11.8	34.7	42.3
mmFormer	24.6	14.8	42.1	58.7
mmFormer + HAGSS	27.3	15.2	42.1	58.7

## Data Availability

The data presented in this study are openly available in GitHub, https://github.com/sqbqamar/HAGSS, which is accessed on 21 March 2026. We have used the Pytorch library for implementation.
